# Tympanic membrane healing in myringotomies performed with argon laser or microknife: an experimental study in rats

**DOI:** 10.1016/S1808-8694(15)31046-6

**Published:** 2015-10-19

**Authors:** Lucio Almeida Castagno, Luiz Lavinksy

**Affiliations:** 1 PhD in Surgery, Otorhinolaryngology - Clínica Dr. Castagno.; 2 Post-Doctorate in Otorhinolaryngology, Head of the Ophthalmology and Otorhinolaryngology Department - Federal University of Rio Grande do Sul.

**Keywords:** argon laser, recurrent acute otitis media, secretory otitis media, wistar rats, myringotomy

## Abstract

Secretory otitis media (SOM) and recurrent acute otitis media (RAOM) may require surgical treatment to proper ventilate the middle ear. Incisional myringotomy is usually done under microscopy with a micro-knife, but it remains patent for just a few days. Recent research indicates that laser assisted myringotomies remain open much longer, allowing middle ear ventilation and healing. **Material and methods:** In this experimental study 34 white, male, adult, Wistar rats, without middle ear disease were submited to anesthesia with ketamine 27 mg/kg and xylazine 2,7 mg/kg. Incisional myringotomy was done on the right ear, while laser myringotomy was done on the left. Myringotomies were evaluated periodically until healing. **Results:** The healing times were equivalent. All myringotomies healed within 10 days. **Conclusion:** Argon laser assisted myringotomy healed just as early on as incisional myringotomy on Wistar rats without middle ear diseases.

## INTRODUCTION

Otitis media bears a rather significant social and economical impact, and it accounts for about 1/3 of pediatric visits because of diseases and for 25% of all oral antibiotic prescriptions in the USA. Tympanocentesis with ventilation tube insertion is one of the most frequently performed surgical procedures in children under general anesthesia. Despite being a simple procedure, it does bear hospital costs and complications may occur. On the other hand, non-treated or poorly treated otitis media does cause sequelae and possibly important functional complications^1^.

Simple tympanocentesis with a microknife incision on the tympanic membrane under the surgical microscope is the standard-classic technique, presenting transitory permeability because the perforation closes in a few days, usually before the middle ear has had a chance to heal^2^. Armstrong^3^ reintroduced the ventilation tube, a method described by Politzer in the late XIX century, aiming at providing longer ventilation to the middle ear. Notwithstanding, the use of non-biological material - ventilation tube - in the tympanic membrane may cause problems, such as otorrhea (12-40% of the cases), progressive tympanosclerosis (48%), tympanic atrophy with retraction pockets (28%), and persistent tympanic perforations^4^.

Tympanocentesis done my microcautery was first described by Saito et al.^5^. The time this perforation takes to heal is longer (15-18 days)^6^, and this fosters middle ear normalization. Since the healing time for laser burns in the skin is very similar to that of cautery cutting, the use of laser in tympanocentesis was introduced in the last 20 years.

CO_2_^7^, ^8^, ^9^, ^10^, ^11^, ^12^ and Neodydio-YAG lasers^13^ used in tympanocentesis were described, and are carried out through systems coupled to the surgical microscope or by oto-probe inserted into the clinical otoscope. These are fast procedures that may be performed in an outpatient basis, under local anesthesia and causing minimal pain, very much popularized in the USA in the early 1998 with the introduction of a CO_2_-laser device coupled to the video-otoscopy system (OtoLAM, ESC Medical Systems, Needham, MA), originally developed at the University Tel-Aviv^14^. It bears the additional advantage of sparing the patients of the tube insertion, thus sparing patients from its possible sequelae.

Soderberg et al.^15^, studied tympanocentesis in Sprague-Dawley rats, and concluded that the microknife incision healing happens in 9, 10, 11 days by hyperplasia of the squamous keratinized epithelium hyperplasia, such epithelium is supported by a highly vascularized connective tissue. After the perforation closes, the epithelium returns to its normal configuration in 1-2 days, while the connective tissue remains thick. In Thermal tympanocentesis, the tympanic vascular supply seems altered, since there are no visible vessels along the malleus process until later in the healing process; histologically, the outer epithelial layer is destroyed much beyond the perforation border, and it is only after 6-9 days that the hyperplasia scar epithelium reaches the collagen in the perforation border; and within 6-9 days afterwards it heals just as it does in microknife incision. The CO_2_ laser tympanocentesis heals similarly to the cautery process, however with less inflammatory reaction and in a slower fashion (18-21 days).

Argon lasers have been used in ophthalmology for many years now. However, we see in the medical literature that its use in tympanocentesis is very limited^16^, ^17^, ^18^, ^19^. The argon laser shares similar characteristics - usually intermediate - to the CO_2_ and Neodymio-YAG laser.

The ultra-structural characteristics of the human tympanic membrane are essentially similar to those of lab animals^20^, ^21^. The middle ear structure has been extensively studied along the last three decades in chinchilla, especially because it presents sizes, shapes and volumes which are very similar to the human ear^22^. The average diameter of the chinchilla tympanic membrane is around 8.32 to 8.53mm, corresponding to about 90% of the human ear drum. On the other hand, the chinchilla middle ear volume is of 1.53ml, while in men it revolves around 2-3ml. Larger mammals may also be used in otologic experiments. Sheep have an average tympanic diameter of 5.3 and 8.2mm, and about 81% of surface equivalent to the human ear drum^23^, ^24^, ^25^.

**Figure 1:**
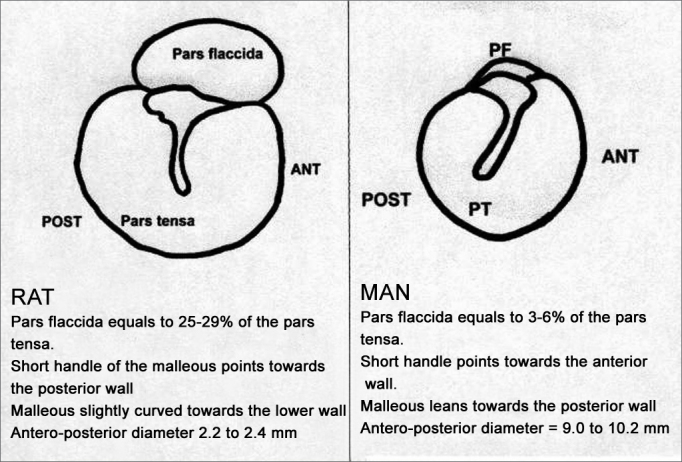
Wistar rat tympanic membrane anatomy compared to the human ear drum.

Studies on the rat middle ear are limited, especially regarding the Wistar lineage. The tympanic diameter found (antero-posterior) varies between 2.2 and 2.4mm. There is a large pars flaccida area, corresponding to almost 1/3 of the pars tensa. The pars flaccida in the live rat can significantly distend with breathing, forming a large bubble, which may occupy almost all the pars tensa in a more labored breathing or when the rat squeaks. The structures of the malleus, incus and stapes are similar to those of human ears. Notwithstanding, both the shape and orientation of the malleus, and most specifically the pars flaccida extension are very distinct (Figure 1). The ossicles are small (about 1/4 of the regular size) and almost totally hidden in the upper tympanic cavity; the mastoid is replaced by a single air cell. Ossicles, facial nerve, stapes and tympanic tensor muscles are very similar to the ones in humans^26^, ^27^. On the other hand, the use of rats as lab animal bears low cost and greater availability than the use of chinchillas.

## MATERIALS AND METHODS

Investigation carried out at the Dr.Castagno’s clinic, in Wistar rats, in 2002-2003, properly approved by the Research Ethics Committee, protocol #01/2002.

Thirty four albino, male, Wistar rats, weighing 250-300g, were anesthetized with a Ketamine 27mg/kg and Xylazine 2.7mg/kg IM solution. The tympanocentesis incision was carried out with a microknife on the right ear (ML-OD), and argon laser tympanocentesis on the left ear (LA-OE). The tympanocentesis was documented by video camera coupled to the rigid Storz endoscope (Hopkins) with 2.7mm diameter. The representative images are printed in a video printer. The follow up exams are done without anesthesia, on the day of the procedure and every 10 days thereafter. We compared the perforation shapes and the healing time for each group. The external auditory meatus was not cleaned with aseptic substance, nor did we use post-operative antibiotic agents.

Incisional tympanocentesis (ML-OD) in rats do not allow the use of human otologic microknives, because the diameter of their tympanic membrane is of 2.2 to 2.4mm. Thus, we decided to use an otologic microperforator, with a very thin and sharp tip in order to carry out the incisional tympanocentesis. After having being anesthetized, the rat is placed in the surgical position. The helper pulls lightly the pinna with a tinny forceps allowing for a straightening of the external meatus, thus facilitating the introduction of the 30° 2.7mm Hopkins rigid endoscope (Storz) all the way down into the acoustic meatus. The rat under an excessively superficial anesthesia contracts its muscles which are adjacent to the auditory meatus, thus preventing the endoscope insertion. Concurrently, we introduce the microperforator a little ahead of the lens, under video control, similar to what we do in nasal endoscopic surgery.

The laser-mediated tympanocentesis (LA-OE), in this study is carried out with an argon laser device PC-EDO (HGM Inc, Salt Lake City, EUA) which allows for a maximum power output of 2.2W, continuous or pulsating, through a small 300 micra microfiber. This microfiber, when placed 1mm short of the target, produces points of 0.55-0.30mm, and with 1.7W we obtain an intensity of 2.405 W/cm^2^ (tip of the microfiber) at 726 W/cm^2^ (1mm short of the microfiber). A pilot study carried out in the tympanic membrane of six rats, having the following parameters chosen for a tympanocentesis, preferable circular in the antero-inferior quadrant were: power 1.9W - duration 0.2 second - distance from the microfiber 1mm - 1 to 2 impacts. The energy density (DE= power x time /surface) in this protocol was of 7.6 J/cm^2^, knowing that with a density above 5J/cm^2^, is possible to vaporize without carbonization.

## RESULTS

The 34 Wistar rats underwent incisional tympanocentesis through a microperforator in the right ear (ML-OD) and argon laser incisions in the left ear (LA-OE) which may be seen in Figure 2ª, Figure 2b, Figure 2c. The incisional tympanocentesis procedures were distributed in three groups, according to their shapes. Radial tympanocentesis, in which the incision extends in a linear fashion between the malleus process and the tympanic annulus (position at 4 to 5 o’clock, considering that the base of the malleus is located at 12 o’clock), was our intent, and it happened in 64.7%. Notwithstanding, due to the very difficulty of the technique in which the microperforator must open the ear drum in less then 1mm in the anterior half-membrane in the rat, there were also triangular-shaped tympanocentesis (20.6%) and broad and irregular tympanocentesis (14.7%). The later, these broad and irregular, which occupy the antero-superior quadrant of the tympanic membrane tend to occur when the microperforator perforates the tympanic membrane in contact with the malleus process and is pulled towards the upper quadrant (located at 1 o’clock), detaching the tympanic segment that is anteriorly linked to the malleus. However, the healing of all these tympanocentesis happened along the first 10 days.

**Figure 2ª:**
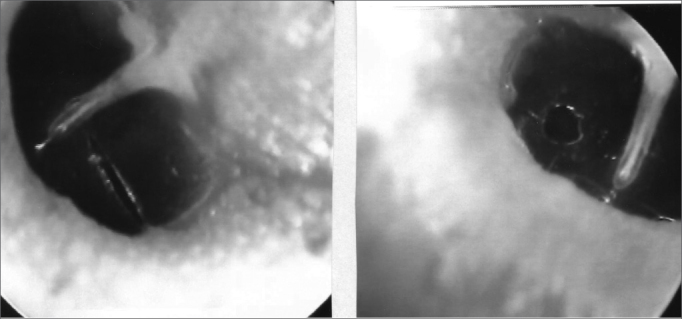
Radial tympanocentesis by microknife (left), and circular by argon laser (right).

**Figure 2b:**
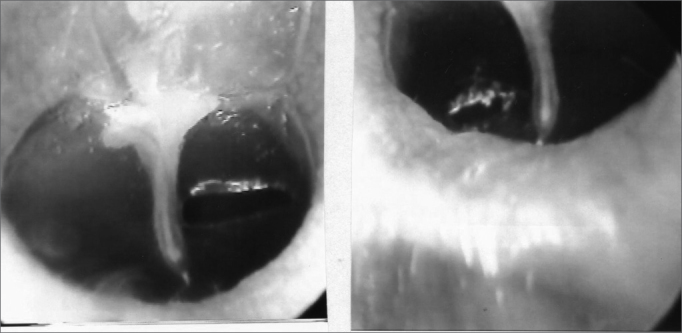
Triangular tympanocentesis by microknife (left), and oval by argon laser (right).

**Figure 2c:**
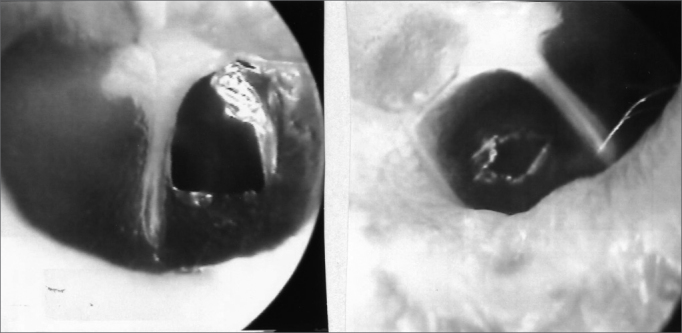
Broad tympanocentesis (left), and irregular by argon laser (right).

The argon-laser-mediated tympanocentesis, from 0.4 to 0.8mm, had three major aspects to them. Circular tympanocentesis (47.1%) happens when the microprobe does not suffer significant shifting and the laser beams hit the same point. Oval-shaped tympanocentesis (47.1%) happens because of adjacent hits. Irregular tympanocentesis happens when the hits are multiple and adjacent (5.9%). All tympanocentesis also healed within 10 days.

## DISCUSSION

Albino Wistar rats have their middle ear very similar to humans’. The auditory meatus is narrower, but it does allow manipulation through videoendoscope in one hand and the micro instrument in the other. The broad availability of these animals, their low cost and easy handling in the laboratory make them a good alternative for experimental otologic microsurgery. Despite all of these, studies about the middle ear anatomy of rats are very limited^28^, ^29^.

The videoendoscopic-controlled tympanocentesis technique developed here is doable after some practice. The endoscope handling is similar to the techniques used for functional endoscopic sinus surgery and, more recently, in tympanoplasties^30^.

The treatment of secretory otitis media has varied along the years. Armstrong^3^, in 1954, popularized the use of ventilation tubes inserted into the tympanic membrane after incisional tympanoplasty with microknives. Tympanocentesis and the insertion of ventilation tubes have become the most common surgical procedure performed in children under general anesthesia, in the USA, adding up to one million procedures per year ^31^. Despite the success of such procedure, there is a certain hesitation from parents and physicians alike in putting these children under general anesthesia, even when it bears minor complications (ventilatory obstructions, long stand recovery, vomits, restlessness), which occur in 9% of the cases, and major complications in 1.9% (laryngospasm and stridor)^32^. The possibility of doing tympanocentesis with the laser, under safely, topical anesthesia and at an outpatient basis in just a few minutes and eventually not needing a ventilation tube would certainly motivate many investigators.

However, the microknife-tympanocentesis healing tends to happen very fast (less than two weeks), and this is not enough to recover most of the secretory otitis media. The tympanocentesis permeability may be extended by the insertion of a ventilation tube, which usually remains for 6-14 months. This time may be excessively long and cause complicatons^33^, ^34^, ^35^. Previous investigations have indicated that healing happens more slowly with the cautery, especially when the incision is done via laser.

In lab animals, thermal tympanocentesis done by a microcautery was studied by Kent and Rhys-Evans^36^ in 50 guinea pigs: 76% healed in 3 weeks, and they were all healed after 6 weeks. There is no reference as to the size of the perforation.

In humans, Goode and Schulz^37^ carried out 2mm thermal tympanocentesis in 10 patients with secretory otitis media: they were all permeable in 3-4 weeks and healed in 6 weeks. Saito et al.5 tried to create permanent tympanocentesis with the cautery: in eight patients, over 25% of the ear drum was perforated and remained patent for 6 months. Perforations smaller than 25% would heal in 3 months and the healing time is directly related to the size of the tympanocentesis. Ruckley and Blair^38^ studied 36 children with secretory otitis media and compared the results of thermal tympanocentesis (3 x 1.5mm) in one ear to the insertion of a ventilation tube in the other: all tympanocentesis done by a cautery healed within 42 days, with an average permeability of 26 days. Potocki and Hoffmann^39^ carried out thermal tympanocentesis (2 to 3mm) in 13 patients undergoing hyperbaric oxygen treatment: 96% of the perforations were permeable in the fifth week and 15% remained patent after 6 months. One has to consider that patients under treatment with hyperbaric oxygen bear important healing impairments (diabetes, vascular peripheral disease, amputations). Amongst us, Wenzel40 performed tympanocentesis with microelectrocautery - Lavinsky-HCPA model - in 83 children: 70.5% were still patent after 90 days.

CO_2_ laser tympanocentesis was initially carried out by Wilpizeski et al.^41^ in 1977, in 40 squirrel monkeys, by removing the whole postero-superior tympanic quadrant. Goode^42^, in 1982, presented a CO_2_ laser tympanocentesis in ten cats and, possible, also the first report in eleven patients. The 1.5 to 2.5mm perforations in cats healed in 3-6 weeks; and 90% of the tympanocentesis (1.5 to 2.5mm in diameter) in human patients healed within 6 weeks. De Rowe et al.14 developed a CO_2_ laser system coupled to an otoscope by fiberoptics and performed 30 tympanocentesis of 1.5 to 2mm in guinea pigs. These remained patent for different amounts of time depending on the amount of energy and the laser duration time: 9.2 days (0.8 Joules/ 0.05s), 15 days (1.2 Joules/ 0.2s) and for 27.4 days (1.6 Joules/ 0.2s). They concluded that the higher the energy output and the longer the time of exposure, the longer the tympanocentesis will remain patent. They also stressed that when they used a fiberoptic microprobe there is laser diffusion on the tip, in such a way that remote structures, such as the promontory or the inner ear do not suffer any damage. In fact, while systems with micromanipulators in surgical microscope have a laser divergence angle of only 3° (and thus do not lose power on the way), the optic microfibers have a 13° divergence, increasing the point of impact according to distance, with significant loss of focus and power. Thus, there is no damage to structures adjacent to the tympanic membrane by the laser that is transmitted through the optic microfibers. Valtonen et al.43 studied the influence of the tympanocentesis shape (from 1.2 to 2.2mm, circular or pear shaped) by CO_2_ laser performed in 18 chinchillas, stating that circular perforations tend to heal faster.

In the present study, Wistar rats underwent argon laser tympanocentesis with 7.6J and 0.2s of duration time. The larger diameter of these tympanocentesis varied from 0.4 to 0.8mm, and the 250 - 300g Wistar rat has an ear drum that varies in diameter from 2.2 to 2.4mm. We did not find any difference among the different modes of tympanocentesis, and they all healed by the tenth day. It is true that these tympanocentesis are smaller that what is advocated in studies involving chinchillas, guinea pigs, monkeys and human patients. However, as we consider the proportion of tympanocentesis in relation to the tympanic membrane diameter we find the following: Wistar rats (0.26), chinchilla (0.25), and in men (0.21). That is, there is no significant proportional difference among the tympanocentesis that are performed in rats. On the other hand, there were no differences in relation to the incisional tympanocentesis carried out by microperforator in the same rats hereby assessed. We still do not know why the argon laser did not present results comparable to those from the CO_2_ laser in tympanocentesis carried out in lab animals.

In humans, CO_2_ laser tympanocentesis seems to have been established as a new and efficient mode of ventilation or drainage for the middle ear, helping to reduce the amount of antibiotics or the need for ventilation tubes in patients with serous otitis media, or even acute otitis media. It is faster and less painful than incisional tympanocentesis, performed under topical anesthesia ^44^, and remains patent for a longer time. Recently, it has also been described in atypical cases, such as Eustachian Tube dysfunction in patients who would travel by plane, hyperbaric oxygen therapy, and mastoiditis^45^. Notwithstanding, Szeremeta et al.7 criticize the excessive emphasis placed on the CO_2_ laser tympanocentesis in both otologic and layman terms, especially when there are no controlled clinical studies in these regards. In a series of 48 incisional tympanocentesis compared to 39 CO_2_ laser-mediated, they concluded that there was no significant reduction in middle ear effusion: 100% of the incisional and 79% of laser tympanocentesis were healed within 16 days. Notwithstanding, recently other authors 46-49 have excitedly reported as to the versatility and efficacy of the CO_2_ laser in keeping the tympanocentesis patent (about 2mm in diameter) for over two weeks in adults and children alike, although results are more limited in allergic patients^50^.
